# The effects of the Competent Manager Training Programme for the self-assessed management and leadership competencies of health and social care managers

**DOI:** 10.1108/LHS-05-2026-0090

**Published:** 2026-10-12

**Authors:** Eevi Karsikas, Anna-Maria Tuomikoski, Eija Hukkanen, Outi Kanste

**Affiliations:** Research Unit of Health Sciences and Technology, University of Oulu, Oulu, Finland; Medical Research Center Oulu, Oulu University Hospital and University of Oulu, Oulu, Finland; Research Unit of Health Sciences and Technology, University of Oulu, Oulu, Finland

**Keywords:** Competence, Health and social care, Management training, Managers, Quasi-experimental study

## Abstract

**Purpose:**

This study aims to describe the effects of the Competent Manager Training Programme on health and social care managers’ self-assessed management and leadership competencies before and after the programme.

**Design/methodology/approach:**

A one-group pretest–post-test design was used. Data were collected from 30 health and social care managers by one public well-being service county in Finland using an electronic self-assessed questionnaire administered before and after an organisation-tailored Competent Manager Training Programme. Data were collected in September 2024 and March 2025. The data were analysed using descriptive statistical methods.

**Findings:**

Management and leadership self-assessed competencies improved significantly after the training programme. The overall mean score increased by 17.2%. The greatest gains were in change management, management of evidence-based practice, human resource management, management of well-being at work and financial management. All competencies showed statistically significant improvement, even development competence. Among background factors, completion of management studies showed the strongest and most consistent associations with competencies. Age, employment type, work experience and number of subordinates were also associated with competencies, whereas gender, education level and budget responsibility were not systematically associated with competencies. However, as the study lacked a control group, causal conclusions regarding the effects of the training programme cannot be drawn.

**Practical implications:**

Training programmes for health and social care managers should be systematically organised to improve managerial self-assessed competence.

**Originality/value:**

This study provides practice-based evidence that an organisation-specific training programme can improve self-assessed management and leadership competencies among health and social care managers.

## 1. Introduction

Health and social care systems face growing pressures, making management increasingly complex (Rojko *et al.*, 2025). Managers in health and social care organisations must balance care expansion, staff shortages, technological advances and Big Data (Ndayishimiye *et al.*, 2022), while nurse leaders confront burnout, workforce gaps and other challenges (Rojko *et al.*, 2025). Additionally, the COVID-19 pandemic has further highlighted the need for organisational and systemic resilience (Ndayishimiye *et al.*, 2022). Effective management and leadership have therefore emerged as critical determinants of health and social care quality and organisational performance, influencing outcomes ranging from resource optimisation to patient well-being (Tasi *et al.*, 2019). Reflecting this, educational and professional development initiatives have increasingly focused on strengthening management and leadership competencies. Health and social care organisations are investing substantial time, funding and effort into management and leadership development to enhance adaptive capacity and long-term sustainability (Alwazzan, 2024; Yemiscigil *et al.*, 2023).

Management and leadership competencies are increasingly recognised as multidimensional constructs, encompassing managers’ personal attributes, clinical expertise and organisational capabilities shaped by their experiences and roles (Chen *et al.*, 2022; Oskarsson and Vik, 2024; Hughes *et al.*, 2022a, 2022b). It is influenced by multiple factors: for example, digital competence depends on individual characteristics, career stage, training and organisational conditions (Laakkonen *et al.*, 2024), while knowledge management is shaped by manager scope and the health and social care context (Karsikas *et al.*, 2025). Competence requirements also vary across managers’ levels, with top managers needing strong strategic and organisational insight, middle managers emphasising operational leadership and first-line managers relying heavily on interpersonal, coaching and motivational competencies to support staff performance and well-being (Rojko *et al.*, 2025; Hedenstrom *et al.*, 2023). Despite these diverse, level-specific competence demands, previous studies show that health and social care managers are often promoted primarily on the basis of clinical expertise rather than formal leadership training, highlighting a persistent gap in structured management and leadership preparation (Hedenstrom *et al.*, 2023).

Health and social care management and leadership competencies are highly contextual (Filomeno *et al.*, 2024; Oskarsson and Vik, 2024), which helps explain why previous studies have identified between 6 and 30 distinct core competencies. Despite this variation, these competencies can be organised into seven overarching competencies: knowledge of the health and social care environment and clinical principles, leadership and people management, professionalism, business and resource management, communication and relationship management, evidence-informed decision-making and continuous improvement and self-leadership (Oskarsson and Vik, 2024; Rojko *et al.*, 2025; Kakemam *et al.*, 2020; Heinen *et al.*, 2019). Because competencies are interrelated, developing one area often reinforces others, and sustained leadership growth requires a holistic and supportive organisational environment that enables managers to apply newly acquired knowledge and competencies in practice (Rojko *et al.*, 2025). In this study, managers’ competence is conceptualised across 12 competencies, including organisational knowledge, quality and safety, human resources, knowledge management, well-being, development, change, crisis management, evidence-based practice, coaching, digital and remote leadership and financial management.

Health and social care managers can develop their skills through multiple avenues, including formal education, in-service training, mentoring, study groups and off-site programmes such as seminars and conferences (Kakemam *et al.*, 2020). Leadership development interventions − especially those incorporating experiential learning, coaching, mentoring, goal setting, 360-degree feedback, shadowing and longitudinal approaches − have been shown to enhance managers’ competence (Alwazzan, 2024; Gunawan *et al.*, 2020; Hedenstrom *et al.*, 2023; Phillipson *et al.*, 2025; Richardson *et al.*, 2023). Programmes tailored to participants′ roles and organisational contexts tend to produce the most sustainable outcomes (Phillipson *et al.*, 2025). For instance, studies report improvements in nurse managers’ authentic leadership, mindfulness and overall competence, although translating learning into practice can remain challenging (Shurab *et al.*, 2024; Chen *et al.*, 2022). Globally, leadership development is increasingly prioritised, with coaching, experiential and project-based approaches linked to improved organisational outcomes (Kingsley-Smith *et al.*, 2024). Programmes that integrate pedagogy with internal or mixed-faculty delivery are generally more effective than content-only or exclusively external training (Kingsley-Smith *et al.*, 2024).

Despite recognition of its strategic importance, empirical evidence on the effects of structured management and leadership training programmes remains limited, particularly across different managers’ levels (Kakemam *et al.*, 2020; Ndayishimiye *et al.*, 2022). This study addresses this gap by evaluating the effects of the Competent Manager Training Programme on health and social care managers’ self-assessed management and leadership competencies using a newly developed, multifaceted Management and Leadership Competencies Scale. This training programme and scale extend previous frameworks by incorporating contemporary competencies such as digital and crisis leadership, emphasising well-being and knowledge management as core leadership responsibilities and integrating coaching-oriented approaches that foster professional growth and organisational learning (Filomeno *et al.*, 2024).

## 2. The study

The study aimed to describe the effects of the Competent Manager Training Programme on health and social care managers’ self-assessed management and leadership competencies before and after the training.

The following research questions (RQs) are proposed:

RQ1.
What are health and social care managers’ self-assessed management and leadership competencies before and after the training programme?

RQ2.
How are background factors associated with managers’ self-assessed competence development?

### 2.1 Design

A quasi-experimental study based on a one-group pretest−post-test was conducted through an electronic questionnaire. This empirical design is used to evaluate the effects of an intervention on a target population without a control group (Polit and Beck, 2018).

### 2.2 Intervention: the competent manager training programme

The intervention involved the Competent Manager Training Programme, implemented between September 2024 and March 2025 in a Finnish well-being services county employing approximately 19,000 staff and 1,000 managers. Well-being services counties are responsible for organising public health and social care services as well as emergency services (Ministry of Social Affairs and Health, 2026). The programme is specifically designed to support managers’ self-assessed competence within public health and social care organisations. Each year, approximately 100 managers participate in the programme, which is designed to enhance the critical management and leadership competencies essential to their roles. Delivered by both internal and external experts from the organisation, the training consists of 43 h, with daily sessions ranging from 3 to 7 h. The programme includes a mix of group, common, remote and in-person meetings. Participants are divided into three groups. A feature of the programme is a 360-degree assessment, which facilitates self-reflection and supports ongoing professional development. Beyond strengthening management and leadership competencies, the training also provides valuable opportunities for peer support and networking within the organisation. Figure 1 shows the schedule and themes for group A training.

**Figure 1.**
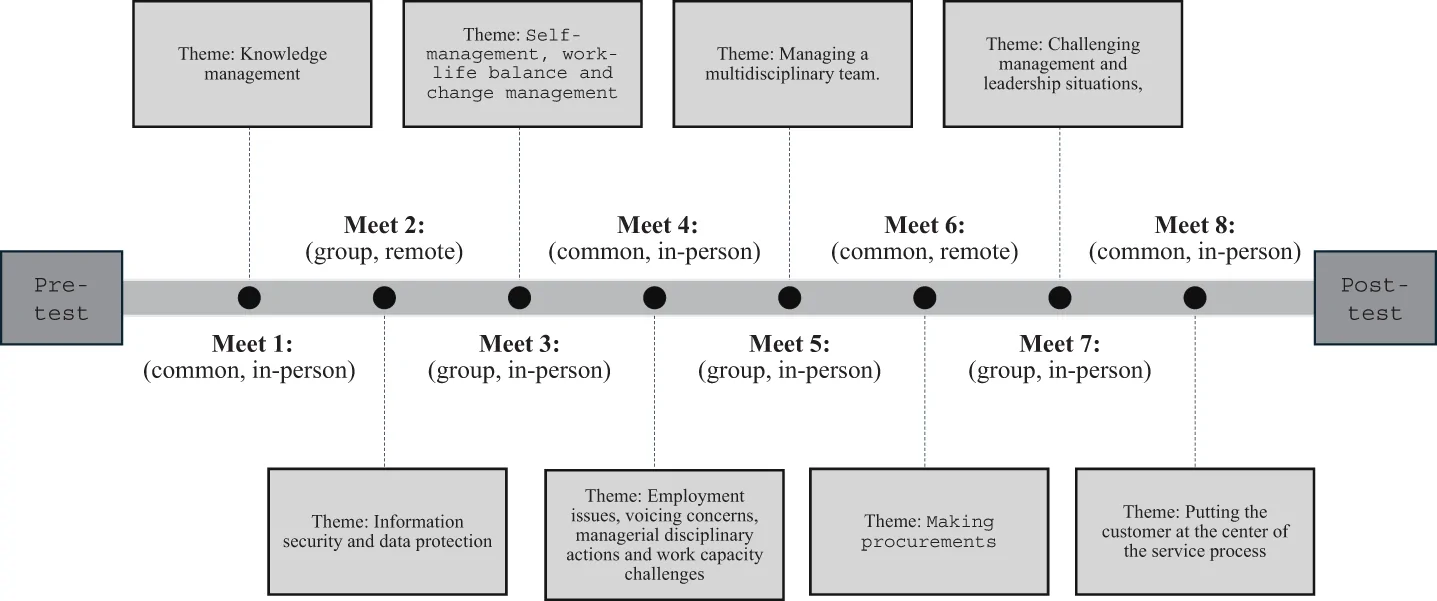
Schedule of the Competent Manager Training Programme

### 2.3 Questionnaire description

To assess health and social care managers’ self-assessed competence, a customised Management and Leadership Competencies Scale developed by the research group was used. The development of the instrument was informed by prior literature reviews (e.g. Almeida *et al.*, 2024; Alwazzan, 2024; Chen *et al.*, 2022; Kakemam *et al.*, 2020; Kingsley-Smith *et al.*, 2024) and an iterative development process carried out in collaboration with managers and leaders from different service areas within a single health and social care organisation. The development team consisted of experts in scale development, nursing professionals, management specialists and experienced educators in management and leadership.

The questionnaire included 17 background questions (see Table 2) and 58 items divided into 12 management and leadership competencies. In addition, it provided an overall measure of a manager’s competence. The reliability of the scale was tested using Cronbach’s alpha to evaluate internal consistency. The Cronbach’s alpha values for the 12 competencies ranged from 0.794 to 0.960, indicating high internal consistency. The overall Cronbach’s alpha for the total scale measuring management and leadership competencies was 0.976. The reliability coefficients (Cronbach’s alpha) for each management and leadership competency were presented in Table 1.

**Table 1.** Management and leadership competencies scale’s Cronbach’s alpha (*n* = 30)

No.	Management and leadership competencies	No. of items	Cronbach’s alpha
1	Knowledge of organisational structures	4	0.854
2	Quality and safety management	5	0.846
3	Human resource management	6	0.898
4	Knowledge management	6	0.879
5	Management of well-being at work	5	0.893
6	Development competence	4	0.848
7	Change management	5	0.902
8	Crisis and exceptional situation management	4	0.958
9	Management of evidence-based practice	4	0.960
10	Coaching leadership	4	0.794
11	Digital and remote leadership	5	0.815
12	Financial management	6	0.917
1–12	Total management and leadership competence	58	0.976

**Source(s):** Authors’ own work

### 2.4 Samples and participants

The study was conducted in Finland, where approximately 200,000 people work in the public health and social care sector. Of these, fewer than 6,000 hold managerial positions, typically serving as immediate supervisors to nurses, the largest professional group in the sector. Due to organisational restructuring and efficiency measures, the number of managers has gradually declined (Finnish Institute for Health and Welfare, 2025).

The survey was conducted online among 105 eligible health and social care managers participating in the Competent Manager Training Programme. The inclusion criteria for the study were as follows: (1) participation in a Competent Manager Training Programme, and (2) acting in managerial roles across all organisational levels. The exclusion criteria were as follows: (1) managers who were not in permanent employment, (2) managers who did not have delegated authority and (3) managers who had participated in training within the past two years.

### 2.5 Data collection

Participants were recruited in September 2024 via an email sent by the first researcher to all health and social care managers (*n* = 105) prior to the start of the Competent Manager Training Programme. The emails provided information about the study, including its purpose, and assured participants of confidentiality and anonymity. The questionnaire was introduced in the email. Participant contact details were provided to the researcher by the training organiser. Electronic informed consent was obtained from all managers who agreed to participate, and they were reminded of their right to withdraw from the study at any time without consequence.

Post-training data collection took place in February 2025, immediately after the final day of the Competent Manager Training Programme, using the same questionnaire. Responses were collected electronically via the Webropol survey tool (Webropol survey tool forerunner, 2026), as online surveys were considered more cost-effective and efficient than paper-based surveys (McMaster *et al*., 2017). Of the 105 participants who attended the training, 63 completed the survey prior to the programme, and 33 completed it afterwards. A total of 30 managers responded to both surveys and consented to participate in the study. Reminder emails were sent before and after the training to encourage participation twice, every two weeks.

### 2.6 Data analysis

The data were divided into two groups: before (T0) and after (T1). The background factors collected at T0 were described using frequencies, percentages, means and minimum and maximum values. Their associations with management and leadership competencies were evaluated using the Mann–Whitney U test (for two-category variables) and the Kruskal–Wallis test (for variables with more than two categories). Changes in competence scores were calculated as percentages. The effects of training on management and leadership competencies were evaluated using the Wilcoxon signed-rank test, which compared values measured before (T0) and after (T1) the training (Munro, 2005). In addition to *p*-values, effect sizes (*r*) were calculated to assess the magnitude of observed changes. In the score, respondents had the option to select “not applicable to my duties.” Only those who had answered at least half of the items of competence were included in the calculation of mean scores. No missing values occurred, as all questionnaire items were mandatory. Statistically, a *p*-value of < 0.05 was considered the threshold of significance (Polit and Beck, 2018). The data were analysed using descriptive statistics and IBM SPSS (version 27.0; IBM, Armonk, NY, USA).

### 2.7 Ethical considerations

The study was conducted in accordance with the Finnish National Board on Research Integrity (TENK, 2021). Permission was obtained from participating organisation, and ethics committee approval was not required, as the study did not involve minors, cause harm or include clinical trials (Medical Research Act 488, 1999). The scale did not require additional authorisation, as it had been previously developed by the research group. Managers provided informed consent by affirmatively answering a mandatory informed consent question at the beginning of the questionnaire. No personally identifiable information was collected. Respondents remained anonymous, and all procedures adhered to General Data Protection Regulation (GDPR, 2018, 2016 / 679). All electronic data collected in the study was securely stored on a protected network drive, accessible only with a username. The research data will be properly destroyed once the results have been reported.

## 3. Results

### 3.1 Characteristics of managers

The study included 30 managers, predominantly women (83%), with an average age of 48 years. Most participants held a master’s degree (70%) and had completed formal management training. The majority worked full-time (73%), with an average of 19 years of experience in the health and social care sector and 4.8 years of managerial experience. On average, the managers oversaw 3.4 units and 44 subordinates; 40% supervised 24 or fewer employees. Budget responsibility varied, with 60% of the managers having no financial responsibility. Participants and dropouts were largely similar in terms of age and gender. However, participants were, on average, more highly educated and more likely to have completed management training, whereas the dropout group consisted of more experienced managers working in organisationally more demanding positions (see Table 2).

**Table 2.** Participants’ (*n* = 30) and dropouts’ (*n* = 34) characteristics

	Participants					Dropouts				
Personal characteristics	*N*	%	Mean	Min.	Max.	*N*	%	Mean	Min.	Max.
*Gender*										
Female	25	83.3				29	85.3			
Male	5	16.7				5	14.8			
Age (years)			48.3	36	65			48.8	29	64
≤39	4	13.3				5	14.7			
40–49	12	40.0				16	47.0			
50–59	13	43.3				10	29.3			
≥60	1	3.4				3	9.0			
*Highest education level*										
Doctoral degree	2	6.7				2	6.0			
Master’s degree	21	70.0				18	52.8			
Bachelor’s degree	5	16.6				13	38.2			
High school or vocational institution	2	6.7				1	3.0			
*Completed management training*										
Yes	21	70.0				21	61.8			
No	9	40.0				13	38.2			
Employment status			50	30	90			50	20	80
Full-time	22	73.3				24	70.6			
Part-time	8	26.7				10	29.4			
Years of experience in health and social care sector			19.0	8.0	41.0			18.3	0	37
≤9	1	3.3				7	20.6			
10–19	13	43.3				9	26.5			
20–29	14	46.7				13	38.2			
≥30	2	6.7				5	14.7			
Years of experience as a manager			4.8	< 1	14			7.9	1	21
≤1	2	6.7				0	0.0			
2–4	17	56.7				16	47			
5–9	7	23.3				6	17.7			
≥10	4	12.3				12	35.3			
Years of experience in current position			2.6	< 1	11			5.0	< 1	21
≤1	8	26.7				8	23.6			
2–3	18	60.0				13	38.2			
≥4	4	12.3				13	38.2			
Number of units managed			3.4	1	32			4.4	1	70
1	15	50				16	47			
2–3	11	36.7				11	32.4			
≥4	4	12.3				7	20.6			
Number of subordinates managed			43.9	0	200			31.5	4	76
≤24	12	40.0				16	47.0			
25–49	8	26.6				10	29.4			
50–99	5	16.7				8	23.5			
≥100	5	16.7				0	0.0			
*Budget responsibility*										
No responsibility	18	60.0				16	47.0			
≤€999,999	6	20.0				9	26.5			
≥€1,000,000	6	20.0				9	26.5			

**Source(s):** Authors’ own work

### 3.2 Change in self-assessed managers’ management and leadership competence

The results of the Wilcoxon signed-rank test revealed statistically significant improvements in all 12 management and leadership competence areas after the intervention (T1) compared to the baseline (T0) (Table 3). The overall mean score of management and leadership self-assessed competence increased from 2.71 (SD = 0.55) to 3.17 (SD = 0.59), representing a 17.2% improvement (*z* = −4.288, *r* = 0.78, *p* < 0.001,). The most notable positive changes were observed in change management (+23.1%, *r* = 0.78, *p* < 0.001), management of evidence-based practice (+22.7%, *r* = 0.45, *p* = 0.014), human resource management (+19.9%, *r* = 0.63, *p* < 0.001), management of well-being at work (+18.9%, *r* = 0.58, *p* < 0.001) and financial management (+20.1%, *r* = 0.67, *p* < 0.001). Although all competencies improved, the smallest relative change was found in development competence (+7.7%, *r* = 0.50, *p* < 0.001). Despite this, all areas reached statistically significant improvement (*p* < 0.05), indicating that the intervention effectively enhanced management and leadership competencies across the different competencies (see Table 3).

**Table 3.** Change in managers’ self-assessed management and leadership competencies (*n* = 30)

		Before T0		After T1			Wilcoxon Signed ranks	Effect size	
N	Management and leadership competencies	Ka. (Kh)	Min−Max	Ka. (Kh)	Min−Max	Change%	*z* =	*r* =	*p*-value
1	Knowledge of organisational structures	3.17 (0.80)	1.75–5.00	3.54 (0.75)	2.25–5.00	+ 11.7	−2.295	0.42	0.022
2	Quality and safety management	2.88 (0.71)	1.00–4.00	3.34 (0.86)	1.00–4.00	+ 16.0	−3.440	0.63	<0.001
3	Human resource management	2.87 (0.76)	1.33–4.00	3.44 (0.76)	2.00–5.00	+ 19.9	−3.430	0.63	<0.001
4	Knowledge management	2.79 (0.65)	1.50–3.83	3.25 (0.70)	1.83–5.00	+ 16.5	−3.708	0.68	<0.001
5	Management of well-being at work	2.85 (0.74)	1.40–4.60	3.39 (0.75)	2.20–5.00	+ 18.9	−3.204	0.58	<0.001
6	Development competence	2.72 (0.70)	1.25–5.00	2.93 (0.54)	1.75–3.86	+ 7.7	−2.766	0.50	<0.001
7	Change management	2.60 (0.71)	1.20–4.00	3.20 (0.74)	2.00–4.80	+ 23.1	−3.756	0.69	<0.001
8	Crisis and exceptional situation management	2.32 (0.92)	1.00–5.00	2.73 (0.87)	1.00–5.00	+ 17.7	−2.692	0.49	<0.001
9	Management of evidence-based practice	2.38 (0.81)	1.00–4.00	2.92 (0.80)	1.50–4.25	+ 22.7	−2.460	0.45	0.014
10	Coaching leadership	2.89 (0.60)	1.50–4.00	3.35 (0.63)	2.00–4.50	+ 15.9	−2.460	0.45	0.002
11	Digital and remote leadership	2.70 (0.63)	1.40–4.00	3.15 (0.75)	1.80–5.00	+ 16.7	−3.072	0.56	<0.001
12	Financial management	2.34 (0.72)	1.00–4.17	2.81 (0.73)	1.17–4.00	+ 20.1	−3.661	0.67	<0.001
1–12	Total management and leadership competence	2.71 (0.55)	1.51–3.76	3.17 (0.59)	2.04–4.21	+ 17.2	−4.288	0.78	<0.001

Note(s):

Scale: 1 = Poor, 2 = Fair, 3 = Good, 4 = Very good, 5 = Excellent

**Source(s):** Authors’ own work

### 3.3 The association between background factors and managers’ management and leadership self-assessed competence

The results showed several statistically significant associations between background factors and management and leadership self-assessed competencies, which varied between measurement points and competencies (see Table 4). The most consistent associations were found with completed management studies, which were significantly associations to several management and leadership competencies at both T0 and T1, particularly competencies 2, 3, 6, 8, 12 and the overall competence score (1–12). Age was also significantly associated with some management and leadership competencies (e.g. competencies 1 and 6). Full-time or part-time employment was statistically significantly associations to certain competencies, particularly competence 11 and the overall score. Years of experience in the health and social care sector and in the current position were associated with specific management and leadership self-assessed competencies, particularly when the duration in the current position was longer. The number of subordinates managed was statistically significantly associated with a few competencies. In contrast, gender, the highest level of education and budget responsibility did not show systematic associations with management and leadership self-assessed competencies.

**Table 4.** The associations between background factors and managers’ self-assessed management and leadership competencies before and after the Competent Manager Training Programme (*n* = 30)

	*Management and leadership competencies*																									
	D1		D2		D3		D4		D5		D6		D7		D8		D9		D10		D11		D12		D13	
Background factors	T0	T1	T0	T1	T0	T1	T0	T1	T0	T1	T0	T1	T0	T1	T0	T1	T0	T1	T0	T1	T0	T1	T0	T1	T0	T1
Gender^b^	0.169	0.051	0.300	0.957	0.448	0.251	0.448	1.000	0.416	0.481	1.000	0.448	0.385	0.875	0.872	0.188	0.059	0.439	0.552	1.000	0.829	1.000	0.327	0.448	0.481	1.000
Age (years)^a^	0.101	*0.033*	0.132	0.137	0.124	0.071	*0.035*	0.460	0.092	0.153	0.254	*0.032*	0.152	0.314	0.201	0.654	0.308	0.663	0.653	0.887	0.129	0.152	0.069	0.264	0.058	0.228
Highest education level^b^	0.993	0.311	0.361	0.120	0.424	0.063	0.748	0.261	0.110	0.420	0.061	0.290	0.235	0.213	0.474	0.162	0.692	0.973	0.203	0.693	0.118	0.113	0.053	0.616	0.272	0.171
Completed management training^a^	0.070	0.050	*0.012*	*0.003*	0.077	*0.011*	0.077	*0.045*	0.077	0.077	0.077	*0.009*	*0.032*	0.050	0.150	0.193	0.236	0.408	0.263	*0.045*	*0.032*	*0.028*	*0.014*	0.050	*0.009*	*0.014*
Full-time or part-time^a^	0.662	0.344	0.909	0.597	0.475	0.202	0.662	0.420	0.170	0.277	0.118	0.219	0.142	0.142	0.256	0.185	0.088	0.320	0.872	0.185	0.078	*0.003*	*0.031*	0.056	0.202	0.050
Years of experience in health and social care sector^b^	0.249	*0.049*	0.419	0.280	0.077	0.235	0.293	0.330	0.383	0.251	0.153	0.188	0.483	0.429	0.564	0.402	0.358	0.516	0.669	0.429	0.584	0.346	0.299	0.494	0.472	0.333
Years of experience as a manager^b^	0.191	0.297	0.211	0.093	0.519	0.305	0.795	0.322	0.263	0.132	0.531	0.345	0.291	0.166	0.591	0.632	0.352	0.712	*0.024*	0.434	0.948	0.567	0.838	0.821	0.625	0.314
Years of experience in current position^b^	0.458	0.223	0.265	0.061	0.654	0.064	0.426	0.251	0.550	0.192	0.209	0.195	0.440	0.462	0.724	0.529	0.121	*0.011*	0.751	0.062	0.479	0.165	0.192	0.185	0.298	0.141
Number of units managed^b^	0.054	0.212	0.487	0.291	0.276	0.818	*0.037*	0.782	0.545	0.986	0.137	0.249	0.526	0.509	0.483	0.958	0.473	0.382	0.770	0.963	0.474	0.440	0.110	0.212	0.097	0.291
Number of subordinates managed^b^	0.866	0.753	0.375	0.665	0.359	0.284	0.648	0.520	*0.028*	0.241	0.647	0.327	0.052	0.270	*0.014*	0.334	0.681	0.090	0.612	*0.025*	0.176	0.572	0.693	0.753	0.159	0.665
Budget responsibility^b^	0.769	0.088	0.528	0.702	0.892	0.965	0.970	0.768	0.482	0.509	0.843	0.916	0.409	0.651	0.551	0.611	0.504	0.628	0.825	0.733	0.825	0.547	0.568	0.339	0.771	0.647

Note(s):

^a^Mann–Whitney U test

^b^Kruskal–Wallis test

D1: Knowledge of organisational structures. D2: Quality and Safety Management. D3: Human Resource Management. D4: Knowledge management. D5: Management of well-being at work. D6: Development competence. D7: Change management. D8: Crisis and exceptional situation management. D9: Management of evidence-based practice. D10: Coaching leadership. D11: Digital and Remote Leadership. D12: Financial Management. D13: Total. T0: Managers’ competencies (baseline). T1: Managers’ competencies (post-Competent Manager Training Programme). Italics values denote statistical significance at the *p* < 0.05 level

**Source(s):** Authors’ own work

## 4. Discussion

This study describes the effects of participating in the Competent Manager Training Programme on managers’ self-assessed management and leadership competencies. The analysis revealed that the training supports the development of management and leadership competencies, both overall and across specific areas. These findings align with the umbrella review by Phillipson *et al.* (2025), which reported that management and leadership development programmes enhance individual competencies. However, they also highlight that evidence for long-term, system-wide effects remains limited. Additionally, the findings highlight the importance of developing a reliable and valid scale for evaluating the competence of health and social care managers. Previous research has consistently highlighted the lack of standardised evaluation methods, making it challenging to assess and compare managers’ capabilities across health and social care settings. By implementing a robust evaluation framework, future studies and training programmes can more accurately assess the effects of competence development initiatives, thereby supporting improved management, leadership and organisational outcomes in health and social care (Ndayishimiye *et al.*, 2022).

The participants of this study are women, with a mean age of 48 years. This reflects a broader national trend in Finland’s health and social care workforce, where the proportion of women is high and the workforce is ageing; this is not an isolated occurrence (Finnish Institute for Health and Welfare, 2025). All respondents were first-line managers, although the programme was also open to middle and senior management. This finding is consistent with previous research suggesting that first-line managers are more active in training programmes, whereas top managers are less frequently involved (Liang and Kakemam, 2025).

*Knowledge of organisational structures* was among the strongest self-assessed competencies before and after training. This finding illustrates that managers are aware of their roles within the organisation and can identify key stakeholders. However, the result is surprising, as previous systematic reviews have shown that guidelines developed to support collaborations primarily focus on clinical competence, whereas deficiencies in management and leadership competencies have been identified as major sources of problems (Vaseghi *et al.*, 2022). After the training, managers rated their competence in *Quality and Safety Management* higher than in *Change Management, even though competence in Change Management* improved more during the programme. This finding contrasts with previous research, which indicated that managers perceived their competence in change management as lower than in quality and safety management (Liou *et al.*, 2022). *Human Resource Management* was the second-best competence after training. This finding illustrates that leaders are aware of their roles within the organisation and can identify key stakeholders. The result is not surprising, as a considerable part of leaders’ work involves, for example, organising staff schedules, fostering a supportive work environment and facilitating opportunities for professional development and training (Oskarsson and Vik, 2024).

Our results showed that, compared to a previous survey assessing managers’ *knowledge management* competence, the participants in our study rated their competence lower before the training but higher after completing it. These findings differ from those of Karsikas *et al.* (2025), who found that managers tended to evaluate their knowledge management competence positively. The difference may indicate that the training increased participants’ awareness of the complexity of knowledge management and helped them better identify their own development needs. According to our results, self-assessed competence in *management of well-being at work* improved by about one in five, representing one of the most significant advancements among the various competencies. Strong competence in leading workplace well-being is essential, as nurse managers play a decisive role in promoting employees’ well-being at work (Almeida *et al.*, 2024). After the training, the weakest area of expertise was *development competence*, which showed the least improvement across all management self-assessed competencies. This finding highlights the need to focus on development competence, as health and social care leaders play a crucial role in organisational performance. Strengthening this competence is essential for leaders to effectively organise work, manage their competencies and implement initiatives that support the organisation’s objectives (Rojko *et al.*, 2025).

According to the results, competence in *crisis and exceptional situation management* was the strongest management self-assessed competence among health and social care managers, both before and after the training. This may be partly explained by the fact that health and social care organisations were compelled to strengthen their capabilities in this area under their leaders’ guidance in response to the COVID-19 pandemic (Ndayishimiye *et al.*, 2022). In contrast, although *Digital and Remote Leadership* is today a key part of managers’ work (Laakkonen *et al.*, 2024), our study showed that competence in this dimension was not rated particularly highly in managers’ self-assessments. One explanation for this may lie in the composition of the respondents: most of those who participated in the survey were women, with an average age of around 50 years. Previous studies have shown that women and managers over the age of 50 tend to have lower levels of digital and remote leadership competence compared to their younger colleagues (Laakkonen *et al.*, 2024).

Our results suggest that participants now feel more confident in using *coaching leadership* after training. This development is significant, as previous research has shown that 100% of coaches sought support in developing their roles and reported gaining a better understanding of themselves and their expertise. Coaching leadership, therefore, not only supports employee growth but also strengthens managers’ own professional identity and capabilities (Richardson *et al.*, 2023). *Financial management*, in contrast, was the weakest area of competence before the training. However, it showed the greatest improvement following the training, indicating that while managers initially struggled in this area, the training was particularly effective in enhancing their financial management self-assessed competence. Similar results have been reported in previous studies, which found financial management to be the weakest competence area (Gunawan *et al.*, 2020) and highlighted managers’ limited confidence in resource management (Liang and Kakemam, 2025). Developing this competence remains essential, as effective resource management underpins organisational efficiency, quality and overall performance (Rojko *et al.*, 2025).

Our results indicate that managers’ self-assessed competence improved across all competencies following the training programme. This finding aligns with previous studies demonstrating a statistically significant positive association between educational interventions and health and social care managers’ performance in areas such as authentic leadership, mindfulness and managers’ competencies (Shurab *et al.*, 2024). While the overall improvement across all competencies averaged 4.1%, certain areas − such as financial management, change leadership and digitalisation − showed markedly greater progress. This suggests that the training’s impact was not uniform across all competence areas but rather concentrated on themes that were particularly emphasised or relevant within the programme. Despite these positive developments, the overall level of managers’ competence achieved remained lower than levels reported in previous studies (Gunawan *et al.*, 2020).

Completed management studies were significantly associated with several competencies, particularly *Development competence, Human resource management, Crisis and exceptional situation management, Financial management* and overall managers’ competence. This underscores the important role of management training in strengthening both management and leadership competencies. Given that only a small proportion of health and social care managers participate annually in the Competent Manager Training Programme, organisations are encouraged to provide more systematic opportunities for competence development. Collaboration between health and social care organisations and educational institutions is essential to ensure access to up-to-date, evidence-based management training that complements internal development practices and supports sustainable growth in managers’ competence.

The analysis indicated that years of experience in the health and social care sector, in managers’ roles or in the current position were not significantly associated with self-assessed management and leadership competencies. Only a few competencies showed statistically significant association, suggesting that while experience may influence specific areas, it is not a consistent predictor of overall competence. This finding supports the notion that health and social care management and leadership competencies are highly contextual (Oskarsson and Vik, 2024). Effective managers’ performance depends not only on experience but also on organisational context, unit complexity and the dynamic challenges of the work environment. Consequently, competence development initiatives should consider these contextual factors to enhance managers’ ability to apply their competence effectively across different situations. Gender and budget responsibility were not systematically associated with management and leadership competencies, which aligns with previous findings indicating that factors such as age may relate more closely to specific competencies, such as digital competence (Laakkonen *et al.*, 2024). Overall, these results complement earlier research highlighting the influence of career paths, job satisfaction and self-efficacy on managers’ competence (Widyana and Handiyani, 2019).

In the Finnish health and social care sector, formal education in management and leadership is available through a variety of routes, including vocational education, universities of applied sciences and universities. However, the contextual nature of the management and leadership competencies identified in this study suggests that organisation-specific management and leadership development programmes could supplement formal education by addressing competence requirements relating to organisational strategy, operating environments and current development priorities. As the needs for management and leadership competencies may vary across organisations and regions, data generated through such programme could support the development of formal management and leadership education by identifying specific competence gaps and emerging educational needs. This could help educational institutions to better align management and leadership curricula with contemporary realities in health and social care management and leadership.

### 4.1 Limitations

This study has certain limitations. Firstly, the study was conducted within a single organisation and focused on management and leadership training tailored to its specific needs, thereby limiting the generalisability of the findings. Secondly, the evaluation was also carried out immediately after the training and without a control group. Although the study design enabled the assessment of changes over time, causal inferences cannot be made, and the observed changes cannot be attributed solely to the training programme. For example, the results may be influenced by changes in maturation, organisational changes or testing. Including a control group and extending the follow-up period would provide clearer evidence that these changes are sustainable. Thirdly, competence was measured through self-report, which may introduce biases such as social desirability or inaccurate self-perception, limitations commonly observed in previous studies assessing managers’ competence (Ndayishimiye *et al.*, 2022; Phillipson *et al.*, 2025).

Fourthly, the small sample size (30 of 105 eligible) and the participation of only frontline managers limit the representativeness of the findings and may not reflect the perspectives of higher-level management. Fifthly, the considerable attrition between the pre-test and post-test measurements may have introduced bias, as participants who dropped out may have differed from those who completed both surveys, for example by being more experienced or holding more senior positions. Consequently, the findings should be interpreted with caution. Finally, as no pilot study was conducted, potential issues related to the clarity, usability or interpretation of the questionnaire may have remained unidentified before data collection.

## 5. Further research

Future research should address the limitations of the current study by including larger, more diverse samples of managers across organisational levels to improve generalisability. Longitudinal designs are recommended to assess the sustained effects of management and leadership training in health and social care, as the long-term effects on patient outcomes and return on investment uncertain (Phillipson *et al.*, 2025). Integrating qualitative methods could provide deeper insight into how managers apply the training in practice, while using control or comparison groups would strengthen causal inference. Furthermore, triangulating self-assessments with objective performance indicators or external evaluations would yield a more comprehensive understanding of managers’ competence development. A notable gap in the existing literature concerns the lack of a shared theoretical foundation in leadership training. Few studies explicitly specify the leadership theories guiding their interventions, resulting in unclear objectives, inconsistent outcomes and programmes that are difficult to compare or align with organisational needs (Phillipson *et al.*, 2025). These observations underscore the importance of adopting theory-informed approaches to management and leadership development. Additionally in future research should compare the impact and effectiveness of organisation-tailored management and leadership development programme with formal management and leadership education programme offered by higher education institutions in health and social care settings.

## 6. Conclusion

This study demonstrates that the developed management and leadership self-assessed competence assessment tool is a reliable scale for evaluating managers’ competence in the social and health and social care sectors. Tailored training programmes, such as the Competent Manager Training Programme, have been shown to enhance multiple management and leadership competencies, particularly change management and the management of evidence-based practice. The findings underscore the critical importance of context-specific and adaptive training initiatives in preparing health and social care managers to address both general and organisation-specific challenges. Moreover, the results highlight the value of ongoing professional development in maintaining and strengthening essential management and leadership competencies, thereby contributing to sustained organisational performance and high-quality patient care. These insights have important implications for the design and implementation of management and leadership development strategies within public health and social care organisations.

### 6.1 Implications for practice

High-level health and social care management and leadership self-assessed competencies are known to directly affect the quality of health and social care and organisational outcomes. The findings of this study offer valuable insights for designing competence-based academic and continuing education programmes for health and social care managers, as well as for developing assessment tools, performance appraisal systems and recruitment strategies. Strengthening health and social care managers’ research and development competencies remains particularly important, as their roles have become less clinical yet increasingly central in facilitating communication among staff and supporting the quality of patient care.

The results also highlight specific management and leadership competencies, particularly development competence and the management of evidence-based practice, as the weakest areas among managers. Given the essential role of these competencies in promoting innovation and ensuring adherence to best practices, future training initiatives should prioritise their development. Moreover, a stronger integration of research-informed content and developmental pedagogies into management education could help bridge the gap between evidence and practice, ultimately contributing to more resilient, learning-oriented health and social care organisations.

## References

[ref001] Almeida, D., Figueiredo, A.R. and Lucas, P. (2024), “Nurseś well-being at work in a hospital setting: a scoping review”, Health and Social Care (Basel, Switzerland), Vol. 12 No. 2, p. 173, doi: 10.3390/healthandsocialcare12020173. PMC10815223 38255062

[ref002] Alwazzan, L. (2024), “Leadership development in medicine: a review”, Cureus, Vol. 16 No. 7, p. e65028, doi: 10.7759/cureus.65028. PMC11334689 39165457

[ref003] Chen, W., Modanloo, S., Graham, I.D., Hu, J., Lewis, K.B. and Gifford, W. (2022), “A mixed-methods systematic review of interventions to improve leadership competencies of managers supervising nurses”, Journal of Nursing Management, Vol. 30 No. 8, pp. 4156-4211, doi: 10.1111/jonm.13828. 36194186

[ref004] Filomeno, L., Forte, D., Di Simone, E., Di Muzio, M., Tartaglini, D., Lommi, M. and Ivziku, D. (2024), “Systematic review and psychometric properties analysis of first-, middle-, and top-level nurse manager′s core competencies instruments”, Journal of Nursing Management, Vol. 2024, p. 2655382, doi: 10.1155/2024/2655382. PMC11918900 40224784

[ref005] Finnish Institute for Health and Welfare (2025), “Health and welfare personnel in the public sector”, available at: Link to the cited article.

[ref006] GDPR (2018), “Guide to the General Data Protection Regulation Information Commissioner′s Office”, Information Commissioner′s Office. [Cited 20 April 2026], available at: Link to the cited article.

[ref007] Gunawan, J., Aungsuroch, Y., Fisher, M.L., McDaniel, A.M. and Marzilli, C. (2020), “Managerial competence of first-line nurse managers in public hospitals in Indonesia”, Journal of Multidisciplinary Healthcare, Vol. 13, pp. 1017-1025, doi: 10.2147/JMDH.S269150. PMC7524169 33061407

[ref008] Hedenstrom, M.L., Spiva, L., Thurman, S., Hale, R.L., Toney, S., Case-Wirth, J., Kairu, M. and Sneha, S. (2023), “Developing and evaluating the effectiveness of a nursing leadership mentoring pilot program”, Nursing Administration Quarterly, Vol. 47 No. 2, pp. 173-181, doi: 10.1097/NAQ.0000000000000557. 36730703

[ref009] Heinen, M., van Oostveen, C., Peters, J., Vermeulen, H. and Huis, A. (2019), “An integrative review of leadership competencies and attributes in advanced nursing practice”, Journal of Advanced Nursing, Vol. 75 No. 11, pp. 2378-2392, doi: 10.1111/jan.14092. PMC6899698 31162695

[ref010] Hughes, R., Meadows, M.T. and Begley, R. (2022a), “AONL nurse leader competencies: core competencies for nurse leadership”, Nurse Leader, Vol. 20 No. 5, pp. 437-443, doi: 10.1016/j.mnl.2022.08.005.

[ref011] Hughes, R., Meadows, M.T. and Begley, R. (2022b), “American organisation for nursing leadership nurse leader core competencies: a framework reaching across the continuum and leadership levels”, The Journal of Nursing Administration, Vol. 52 No. 12, pp. 629-631, doi: 10.1097/NNA.0000000000001221. 36409252

[ref012] Kakemam, E., Liang, Z., Janati, A., Arab-Zozani, M., Mohaghegh, B. and Gholizadeh, M. (2020), “Leadership and management competencies for hospital managers: a systematic review and best-fit framework synthesis”, Journal of Healthcare Leadership, Vol. 12, pp. 59-68, doi: 10.2147/JHL.S265825. PMC7383104 32801985

[ref013] Karsikas, E., Meriläinen, M., Koivunen, K. and Kanste, O. (2025), “Health and social care managers′ self-assessed competence in knowledge management: a descriptive cross-sectional study”, Journal of Advanced Nursing, Vol. 81 No. 2, pp. 787-797, doi: 10.1111/jan.16237. PMC11729214 38752616

[ref014] Kingsley-Smith, H., Farrier, C.E., Foran, D., Kotze, K., Mahtani, K., Short, S., Scott, A.M. and Lyons, O. (2024), “Leadership development programmes in health and social care research: a systematic review, meta-analysis and meta-aggregation”, BMJ Leader, Vol. 8 No. 4, pp. 283-292, doi: 10.1136/leader-2024-000976. 38991736

[ref015] Laakkonen, N., Jarva, E., Hammarén, M., Kanste, O., Kääriäinen, M., Oikarinen, A. and Mikkonen, K. (2024), “Digital competence among health and social care leaders: a mixed-methods systematic review”, Journal of Nursing Management, p. 8435248, doi: 10.1155/2024/8435248. PMC11919023 40224897

[ref016] Liang, Z. and Kakemam, E. (2025), “Identifying competency development needs of hospital managers in Iran: a national survey”, BMC Medical Education, Vol. 25 No. 1, p. 122, doi: 10.1186/s12909-025-06721-x. PMC11762520 39856660

[ref017] Liou, Y.F., Lin, P.F., Chang, Y.C. and Liaw, J.J. (2022), “Perceived importance of competencies by nurse managers at all levels: a cross-sectional study”, Journal of Nursing Management, Vol. 30 No. 3, pp. 633-642, doi: 10.1111/jonm.13545. 34989045

[ref1031] McMaster, H.S., LeardMann, C.A., Speigle, S. *et al*. (2017), “An experimental comparison of web-push vs. paper-only survey procedures for conducting an in-depth health survey of military spouses”, BMC Medical Research Methodology, Vol. 17, pp. 1-9, doi: 10.1186/s12874-017-0337-1. PMC5405460 28441932

[ref018] Medical Research Act 488 (1999), “Given in helsinki 01.11.1999. [cited 20 april 2026]”, available at: Link to the cited article.

[ref019] Ministry of Social Affairs and Health (2026), “Wellbeing services counties will be responsible for organising health, social and rescue services”, available at: Link to the cited article.

[ref020] Munro, B. (2005), Statistical Methods for Health and Social Care Research, (5th ed.), Lippincott Williams and Wilkins.

[ref021] Ndayishimiye, C., Sowada, C., Dyjach, P., Stasiak, A., Middleton, J., Lopes, H. and Dubas-Jakóbczyk, K. (2022), “Associations between the COVID-19 pandemic and hospital infrastructure adaptation and planning-A scoping review”, International Journal of Environmental Research and Public Health, Vol. 19 No. 13, p. 8195, doi: 10.3390/ijerph19138195. PMC9266736 35805855

[ref022] Oskarsson, I.M.L. and Vik, E. (2024), “Suggesting a holistic framework for understanding health and social care services leadership competence – a critical interpretive synthesis”, Leadership in Health Services, Vol. 37 No. 5, pp. 99-129, doi: 10.1108/LHS-08-2023-0059. PMC11348838 38619933

[ref023] Phillipson, J., Pinto, A.C., Kingsley-Smith, H., Krachler, N., McGivern, G. and Lyons, O. (2025), “Leadership training in health and social care: a systematic umbrella review”, BMJ Leader, Vol. 9 No. 4, doi: 10.1136/leader-2025-001269. PMC12772614 40527607

[ref024] Polit, D.F. and Beck, C.T. (2018), Essentials of Nursing Research: appraising Evidence for Nursing Practise, (9th ed.), Wolters Kluwer Health.

[ref025] Richardson, C., Wicking, K., Biedermann, N. and Langtree, T. (2023), “Coaching in nursing: an integrative literature review”, Nursing Open, Vol. 10 No. 10, pp. 6635-6649, doi: 10.1002/nop2.1925. PMC10495732 37365717

[ref026] Rojko, L., Kvas, A. and Stare, J. (2025), “Leadership competencies in public health: implications for nursing leadership – a scoping review”, Leadership in Health Services, Vol. 38 No. 2, pp. 211-229, doi: 10.1108/LHS-06-2024-0053.

[ref027] Shurab, W.M.E., Ibrahim, S.A.E., Abdelaalem, M.M., Gabal, S.A.A. and Abdelhady, T.R.M. (2024), “Effect of authentic leadership and mindfulness educational program on nursing managers′ competencies: a quasi-experimental study”, BMC Nursing, Vol. 23 No. 1, p. 342, doi: 10.1186/s12912-024-01976-z. PMC11106989 38773603

[ref028] Tasi, M.C., Keswani, A. and Bozic, K.J. (2019), “Does physician leadership affect hospital quality, operational efficiency, and financial performance?”, Health Care Management Review, Vol. 44 No. 3, pp. 256-262, doi: 10.1097/HMR.0000000000000173. 28700509

[ref029] TENK (2021), “The ethical principles of research with human participants and ethical review in the human sciences in Finland”, Integrity TENK guidelines, 3/2019, 2nd. [Cited 20 April 2026], available at: Link to the cited article.

[ref030] Vaseghi, F., Yarmohammadian, M.H. and Raeisi, A. (2022), “Interprofessional collaboration competencies in the health system: a systematic review”, Iranian Journal of Nursing and Midwifery Research, Vol. 27 No. 6, pp. 496-504, doi: 10.4103/ijnmr.ijnmr_476_21. PMC9881554 36712303

[ref031] Webropol survey tool forerunner (2026), “Webropol – A forerunner for leading with information”, available at: Link to the cited article.

[ref032] Widyana, R. and Handiyani, H. (2019), “Self-efficacy and organizing competency of head nurse dominant factors determining of nursing job satisfaction implemented in two hospitals in Riau province”, Enfermería Clínica, Vol. 29, pp. 625-628.

[ref033] Yemiscigil, A., Born, D. and Ling, H. (2023), “What makes leadership development programs succeed?”, Harvard Library, available at: Link to the cited article.

